# Local health workers’ perceptions of substandard care in the management of obstetric hemorrhage in rural Malawi

**DOI:** 10.1186/1471-2393-13-39

**Published:** 2013-02-15

**Authors:** Jogchum Jan Beltman, Thomas van den Akker, Dieudonné Bwirire, Anneke Korevaar, Richard Chidakwani, Luc van Lonkhuijzen, Jos van Roosmalen

**Affiliations:** 1 Thyolo District Health Office, Ministry of Health, Thyolo, Malawi; 2 Department of Obstetrics, Leiden University Medical Centre, Leiden, The Netherlands; 3 Médicins Sans Frontières, Thyolo, Malawi; 4 Department of Obstetrics, University Medical Centre Groningen, Groningen, The Netherlands; 5 Department of Gynecologic Oncology, University of Toronto, Toronto, Canada; 6 Department of Medical Humanities, EMGO Institute for Health and Care Research, VU University Medical Centre, Amsterdam, The Netherlands

## Abstract

**Background:**

To identify factors contributing to the high incidence of facility-based obstetric hemorrhage in Thyolo District, Malawi, according to local health workers.

**Methods:**

Three focus group discussions among 29 health workers, including nurse-midwives and non-physician clinicians (‘medical assistants’ and ‘clinical officers’).

**Results:**

Factors contributing to facility-based obstetric hemorrhage mentioned by participants were categorized into four major areas: (1) limited availability of basic supplies, (2) lack of human resources, (3) inadequate clinical skills of available health workers and (4) substandard referrals by traditional birth attendants and lack of timely self-referrals of patients.

**Conclusion:**

Health workers in this district mentioned important community, system and provider related factors that need to be addressed in order to reduce the impact of obstetric hemorrhage.

## Background

Postpartum hemorrhage (PPH) is one of the main causes of maternal mortality worldwide [1-3]. In Malawi, a low-income country in sub-Saharan Africa with a very high maternal mortality ratio, obstetric hemorrhage accounted for 14% of all maternal deaths in 2001 [4].

A retrospective review of obstetric hemorrhage in Thyolo District, Malawi, revealed 133 cases of hemorrhage among 3085 hospital deliveries (43.1 per 1000 deliveries). Six women died as a result of bleeding leading to a case fatality rate of 4.5%, which is far more than the 1% that is deemed acceptable by the World Health Organization [5,6]. Several substandard care factors in the management of obstetric hemorrhage were identified from medical records. These included insufficient monitoring of pregnant women, performing unnecessary cesarean sections on demised fetuses and inconsistent use of uterotonics [6].

Because risk factors for hemorrhage such as prolonged labor and grand multiparity are common in many low-resource settings, health workers in these areas face high numbers of obstetric hemorrhages in their clinics. However, they often have limited means to address this burden. It is rare that perceptions about substandard care held by these health workers are included in accounts of the quality of obstetric care.

In recent years, several reports have given recom-mendations for the improved management of obstetric hemorrhage [7,8]. The conclusions of these reports are often based on the external investigators’ point of view, and rarely take into account local capacity. In the present study, we assessed which factors were perceived to be associated with facility-based obstetric hemorrhage by health workers in Thyolo District.

## Methods

### Study setting and population

This study was conducted in September 2006 in Thyolo, a rural district in southern Malawi, with a population of approximately 570 000 in 2005. Health facilities in the district consisted of a public hospital (Thyolo District Hospital) and 26 smaller government, mission and private health facilities (usually referred to as ‘health centers’). Peripartum care at the facility level is provided by nurse-midwives and non-physician clinicians (‘medical assistants’ and ‘clinical officers’), who work independently or with limited supervision of a physician. Medical assistants provide general primary care and are often based in peripheral health centers, while clinical officers also perform secondary level procedures, including caesarean section, and usually work in district hospitals. In addition, more than 100 traditional birth attendants operate outside the formal sector [9]. The total number of deliveries in this district was estimated at 28 500 (birth rate 50 ‰), half of which occurred in formal health facilities [10,11], where workload is immense. Up-to-date obstetric protocols are not available. At the district hospital, 3 085 deliveries took place in 2005. The cesarean section rate was 13% and the antenatal HIV prevalence 22% [12]. A prevention-mother-to-child-prevention (PMTCT) program in Thyolo was run by Medicins sans Frontieres (MSF) Belgium at time of the study.

### Focus group discussions

Three separate focus group discussions (FGDs) were carried out with a total of 29 respondents (8 clinical officers, 14 nurse-midwives and 7 medical assistants). Respondents were purposely sampled to source information from health care providers dealing with obstetric care on a routine basis and each group included health care providers from both district hospital and peripheral health centers. Topics that were discussed included causes and clinical management of obstetric hem-orrhage, as well as provider- and patient-related factors, with a focus on obstetric care providers’ knowledge and skills.

The FGDs were led by a social communicator (who encouraged participants asking questions, exchanging anecdotes and commenting on each other's experiences and points of view) and one medical intern specifically trained for this purpose. Discussions were held at the district hospital and conducted in the English language, which is spoken by most health professionals as a second language. All discussions were audio-recorded and transcribed verbatim. Using the open code technique, a content analysis was done. This technique implies a systematic classification process of coding and identifying themes or patterns or quotes until saturation is reached and no new information is found [13]. Quotes were drawn to indicate similarities and differences between respondents. Consent was sought before participation and all data were depersonalized so that reports could not be traced to individuals.

## Results

Four themes were identified: lack of materials (1), lack of human resources (2), inadequate clinical skills among available personnel (3) and inadequate referrals, subdivided into self-referrals, and referrals from TBAs and peripheral clinics (4). Characteristics (age and work experience) of the 29 participants are listed in Table 1.

**Table 1:** Characteristics of respondents

	**Mean age (years)**	**Work experience (years)**
Clinical officers (n= 8)	32.6	5.6
Medical assistants (n= 7)	30.8	6.7
Nurse midwives (n= 14)	30.4	6.8

Limited availability of basic supplies was felt to be a major cause of substandard obstetric care in health centers and hospital. *“Sometimes you find that you are lacking the IV fluids” (clinical officer). “Sometimes Pitocin (oxytocin, JB) gets out of stock”. “It is another challenge when ergometrine gets out of stock” (nurse-midwife). “Most of the time everything is out of stock. We don’t have enough for the whole month” (medical assistant).*

Most participants identified a lack of human resource as a major barrier to improving the quality of obstetric care. *“So we can say, we have problems in managing these hemorrhages, because not all the time people are there to assist you. We assist one another, it is not only a clinician or a nurse who manages PPH, but if there are other complications you also need theatre people. So for those theatre people to come, that is so time consuming” (clinical officer) “Another major cause of maternal death is lack of enough skilled workers in this midwifery practice” (nurse midwife).*

But perhaps more interestingly, many participants highlighted the inadequate clinical skills of available health workers. *“If someone is not well equipped with the knowledge and skills necessary to save lives, then we don't expect that one to perform wonders” (clinical officer).* Most respondents had not attended any refresher training since they started their professional careers (in some cases more than ten years before). Lack of training was seen as a major shortcoming. *“It was a long time ago. It must be, if it is not four years ago, something like that, at that time almost every midwife was trained” (nurse midwife).* Several health workers assigned blame for inadequate clinical skills among the workforce to policy makers whom, they reported, gave low priority to obstetric training. *“I have been working for over ten years and have had a refresher course only once. You can imagine, every time they (policy makers, JB) talk about maternal death instead of giving health workers some more training on PPH. They just talk. I think most of the attention the government spends to management of HIV/AIDS rather than maternal death” (medical assistant).*

Lack of both TBA- or patient-related timely referrals was believed to be an important factor associated with morbidity and mortality from obstetric hemorrhage. There was a widespread belief among respondents that TBAs deliberately kept patients too long within their care without referring them. They were said to use dangerous amounts of traditional herbal oxytocics and to persuade women to deliver outside formal clinics by understating the risk of complications. *“Sometimes TBAs take African medicine to keep the patient, meaning to deliver quickly, not knowing about big baby or transverse lie. And if that medicine makes tonic contraction, it can rupture the uterus” (nurse midwife). “The main challenge is that most of the PPH that I have encountered come from the TBA. The TBA keeps the patient too long for them to get something. After realizing there is PPH, they refer that patient to the health center. But when they refer the patient, it is still too late” (medical assistant). “TBAs are not trained to do vaginal examination. So they do encourage patients to push while the cervix is not fully dilated. So this could be a risk for cervical tear” (nurse midwife).*

Lack of timely self-referral of patients or delay in the decision to seek care (the first phase of delay [14]) was also thought to be an important reason. *“In obstructed labor, they think someone has bewitched her, so that the women do not deliver. So they think if they come to hospital, they still will not deliver.”* Women’s negative perceptions of the quality of health care in formal facilities and their fear of being stigmatized by health care personnel were recognized by most respondents as contributing factors to a delay in seeking care. *“They are afraid of caesarean section; they say they will die at the theatre table”.* Jehovah’s witnesses, a prominent religious group in the district, were found to endanger their own health by refusing blood transfusion*. “Another challenge in the management of PPH is the fact that we are people of different denominations. Some of us refuse the replacement of blood”.*

Despite the social and cultural importance of child bearing in African society, unwanted pregnancies are a source of problems within the family. This holds true for adolescents who fall pregnant and where resorting to abortion is commonly their only choice to avoid judgment from their family and community. *“Abortions, unwanted pregnancies. Even the young school girls are dying, because of abortions or criminal abortions”.*

Delay in health care seeking behavior was thought to sometimes have its origin in health promotion efforts. *“We encourage people to practice family planning, so if people have more than four deliveries, they do not come to health facilities for health services, because they fear they can be influenced and as a result they opt for alternative means of delivery. They deliver at home” (clinical officer).*

Health care worker-related reasons were mainly comprised of attitude problems. Most respondents recognized and rejected unkind behavior towards patients and acknowledged that this may affect care-seeking behavior. *“As a result, women choose probably to be assisted or to deliver at the TBA or at home” (nurse-midwife).*

Many of the respondents blamed this conduct on excessive workload. “*Because of this reduced number of midwives, many of them are exhausted, because there is a lot of work to be done. And they lose their temper in that process, they are unable to control their attitudes and sometimes they talk maybe some words which are not good. Words which can make a woman probably feel belittled. And this can also in turn cause reduced utilization of the facility” (nurse midwife). “How can one midwife fight against ten laboring patients, even more than ten per day. It is very, very tiresome. You lose your temper” (nurse midwife). “Yet if you are calling for help, nobody is coming, so it is difficult for that PPH to be managed as proper as it can be” (nurse midwife).*

## Discussion

Our study revealed a number of important community- and provider-related operational and cultural barriers that have resulted in program adaptations such as the introduction of obstetric audit on a regular basis, enhanced provision of on-the-job training, increased ward supervision and improvements in the availability of human and material resources in a number of health clinics [15,16].

Firstly, the chronic lack of materials and supplies (especially iv-fluids and oxytocin) that is experienced within the district health facilities is not particular for this district. On the contrary; this same picture appeared in a national assessment of emergency obstetric care (EmOC) in Malawi in 2005. It showed low coverage of basic EmOC (intended to be offered by health centers) and therefore poor usage, and poor quality as evidenced by high case fatality rates [17]. Efforts were made with help of Medecins Sans Frontières Belgium, Malawi mission, Thyolo project (MSF) to assist with supplies in case of emergency. This dependence on external support is illustrative of a struggling public health system.

Secondly, referrals of obstetric patients from TBAs seemed to bring about concerns among nearly all participants. Many examples were given of patients that were transferred to health facilities with considerable delays. Reluctance of TBAs to refer in a timely manner and absence of basic skills among TBAs were openly denounced. These findings indicate a sharp contrast between the opinions of health workers about TBAs on the one hand, and the high levels of acceptability and trust among pregnant women on the other. Survey data showed an increase in proportion of deliveries attended by TBAs between 1992 and 2000 [18]. During a yearly training for TBAs organized in the district, increased emphasis was put on indications for referral and risk factors for antepartum- and postpartum hemorrhage. TBAs were also provided with necessary items to practice clean and safe deliveries. A similar FGD involving traditional birth attendants was conducted separately.

Patient-related factors such as fear of dying during cesarean section or fear of being influenced by information about family planning were also mentioned as barriers hindering adequate maternal care.

Provider-related factors were also identified. Overbearing attitudes of health care providers towards patients, a poorly functioning transport system with inefficient use of ambulances and delays at the hospital level in assessing emergency cases by clinicians and late arrival of hospital theatre staff for emergency procedures were brought up frequently. Some of these issues were also identified during separate audit sessions and actions were successfully undertaken to address these [15]. For example, the delay in assessing emergency cases by clinicians was reduced by setting up a night station for clinicians in the emergency department, so that they could stay on-site over night.

Thirdly, the persistent lack of human resources plays a major role in managing obstetric hemorrhage. Understaffed health care systems must manage many pressing health problems. Malawian vacancy rates in critical healthcare positions are very high - in 2004, 68 percent of doctors, 58 percent of nurses and 32 percent of clinician positions remained unfilled [19]. With such gross understaffing, inadequate referrals by both providers and patients themselves may be understandable, but should certainly not always be taken for granted and rendered acceptable.

Fourthly, respondents put emphasis on a lack of training in obstetric care. Many of them mentioned the absence of up-to-date protocols in health facilities. Many had not attended any skills training for four or five years. Data from maternal mortality reviews in Malawi indicate a lack of obstetric life-saving skills. Substandard care accounted for 60.5% of all maternal deaths [20]. Obstetric hemorrhage as cause for maternal deaths accounted for 25.6% [20]. From 2006 onwards a selected group of health care workers (around ten persons each year) received the advanced life-support in obstetrics (ALSO) training course, which was facilitated twice a year. Items covered in this course included management of postpartum hemorrhage, shoulder dystocia and vacuum delivery.

## Conclusions

In many areas of the world, safe delivery for all women remains an illusion. This study highlights provider/service-related health care deficiencies such as lack of privacy, neglect and improper attitude towards patients. These deficiencies impact on care seeking behaviour of patients and their analysis adds importantly to the concept of substandard care. We therefore think that including the observations of health care workers can be of added value.

### Ethical approval

Verbal approval was obtained from the National Health Sciences Research Committee from the Ministry of Health, Malawi, which ruled that formal approval was not necessary for this type of study. In addition, the National Health Sciences Research Committee as well as the District Health Office of the Ministry of Health ruled that written consent was not necessary for this type of operational research, which should arguably be routine practice in any district hospital in order to monitor clinical performance.

The District Health Office of the Ministry of Health took part in the study design and ensured that the study was performed conform national guidelines. This study did not involve any patients and none of the results can be traced back to individual health workers who participated in the FGDs.

## Competing interests

The authors declare that they have no competing interests.

## Authors’ contributions

JJB and DB designed the study protocol; AK, RC and JB implemented the study and were involved with data analysis; TvdA, LvL, JvR and AK contributed to the data analysis; JB and TvdA wrote the first draft of the manuscript and handled the different editions and revisions. All authors read and approved the final manuscript, and contributed significantly to the intellectual content.

## Pre-publication history

The pre-publication history for this paper can be accessed here:

http://www.biomedcentral.com/1471-2393/13/39/prepub
